# Comparing terrestrial laser scanning and unmanned aerial vehicle structure from motion to assess top of canopy structure in tropical forests

**DOI:** 10.1098/rsfs.2017.0038

**Published:** 2018-02-16

**Authors:** Sabina Roşca, Juha Suomalainen, Harm Bartholomeus, Martin Herold

**Affiliations:** 1 Laboratory of Geo-Information Science and Remote Sensing, Wageningen University, Droevendaalsesteeg 3, 6708 PB Wageningen, The Netherlands; 2 Finnish Geospatial Research Institute, National Land Survey of Finland, Geodeetinrinne 2, 02430 Masala, Finland

**Keywords:** terrestrial LiDAR, terrestrial laser scanning, structure from motion, unmanned aerial vehicle, top of canopy, tropical forest

## Abstract

Terrestrial laser scanning (TLS) and unmanned aerial vehicles (UAVs) equipped with digital cameras have attracted much attention from the forestry community as potential tools for forest inventories and forest monitoring. This research fills a knowledge gap about the viability and dissimilarities of using these technologies for measuring the top of canopy structure in tropical forests. In an empirical study with data acquired in a Guyanese tropical forest, we assessed the differences between top of canopy models (TCMs) derived from TLS measurements and from UAV imagery, processed using structure from motion. Firstly, canopy gaps lead to differences in TCMs derived from TLS and UAVs. UAV TCMs overestimate canopy height in gap areas and often fail to represent smaller gaps altogether. Secondly, it was demonstrated that forest change caused by logging can be detected by both TLS and UAV TCMs, although it is better depicted by the TLS. Thirdly, this research shows that both TLS and UAV TCMs are sensitive to the small variations in sensor positions during data collection. TCMs rendered from UAV data acquired over the same area at different moments are more similar (RMSE 0.11–0.63 m for tree height, and 0.14–3.05 m for gap areas) than those rendered from TLS data (RMSE 0.21–1.21 m for trees, and 1.02–2.48 m for gaps). This study provides support for a more informed decision for choosing between TLS and UAV TCMs to assess top of canopy in a tropical forest by advancing our understanding on: (i) how these technologies capture the top of the canopy, (ii) why their ability to reproduce the same model varies over repeated surveying sessions and (iii) general considerations such as the area coverage, costs, fieldwork time and processing requirements needed.

## 1. Introduction

Forest ecosystems are an important global resource playing key roles in both the environment and the economy. In the context of sustainable development and climate change mitigation, tropical forests are a major focus for research due to the role they play in the global carbon cycle, and recently, in climate mitigation policies through REDD (reduced emissions from deforestation and degradation) [1]. Remote sensing techniques are increasingly valued by ecologists for the unique perspective they offer to describe ecosystem states and dynamics [2]. They have proven to be successful when it comes to understanding forest structure, from plot-scale measurements using terrestrial laser scanning (TLS) [3–9], to meso-scale (1–100 km^2^) using aerial laser scanning [10–14] and aerial imagery [15–17], and up to global-scale perspective from satellite imagery [18,19], radar [20] and light detection and ranging (LiDAR) [21,22].

In recent years, TLS and airborne laser scanning (ALS) have attracted much attention from the forestry community as rapid and efficient tools for quantifying forest parameters. Although researchers confirm that ALS is an adequate method to estimate canopy height in coniferous and deciduous forests [11,12,23,24], there are few studies that have evaluated canopy height with ALS in tropical forests [25–29]. In turn, TLS is capable of acquiring levels of detail far beyond what ALS is capable of [30]. The high-resolution capability offers many exciting opportunities for vegetation research, and several studies [3–9] have explored TLS applications in measuring forest structure and tree parameters.

More recently, the usage of small unmanned aerial vehicles (UAVs) has increased dramatically. This has been made possible by advancements in technology such as the availability of accurate and miniature global navigation satellite systems, inertial measurement units, smaller and lighter batteries, and high-quality consumer digital cameras. With the advent of new algorithms, such as the Scale Invariant Feature Transform (SIFT), that can directly georeference and rectify the imagery with only low accuracy camera positions, UAVs also found their use as remote sensing tools [31]. These platforms can be customized and equipped with different sensors [32]. For example, Wallace *et al*. [33] are using a UAV equipped with laser scanning sensors, while in other studies [31,34–38], UAVs equipped with digital cameras are used.

At present, UAVs equipped with digital consumer cameras are considerably cheaper compared with UAVs equipped with laser scanning sensors. The large sets of overlapping digital photographs, taken from different locations, can be automatically post-processed to geometrically precise three-dimensional point cloud datasets [35], using computer vision structure from motion (SfM) algorithms.

The goal of this study is to provide a comparison between UAV-SfM and TLS in assessing the top of canopy structure, at a study site in the tropical forest of Guyana. We assessed (i) how different features in forest structure affect the quality of the top of canopy modelled with the two technologies, (ii) how accurately forest change is captured by the two technologies and (iii) if, over repeated surveying sessions, the TLS and the UAV-SfM derived point clouds render the same top of canopy model (TCM).

## 2. Material and methods

### 2.1. Study site

The fieldwork data were acquired in November 2014, in a rectangular plot of 30 × 40 m, in a tropical forest of central Guyana (6°2′10′ N, 58°41′55′ W), south of Bartica. The climate is tropical, with rainfall of 2764 mm yr^−1^ at Bartica Station (hydromet data 1958–1975) [39], and two distinct dry periods: January to March and August to September [40]. The mean daily temperature over the year is 25°C. The region containing the study area is covered by dry evergreen forest, which is found almost exclusively on well-drained, bleached white sands [39]. In the region, the average tree density (with a diameter at breast height greater than 10 cm) is 650 trees ha^−1^, while the mean number of species is 68 species ha^−1^ [41].

### 2.2. Equipment and data collection

The fieldwork data, both TLS and UAV, were acquired over the same plot, at two different periods of time that were 3 days apart. Between the two sessions, a tree was harvested in the plot area to study the sensitivity of the two modelling technologies to change.

#### 2.2.1. Terrestrial laser scanning data collection

**Figure 1.**
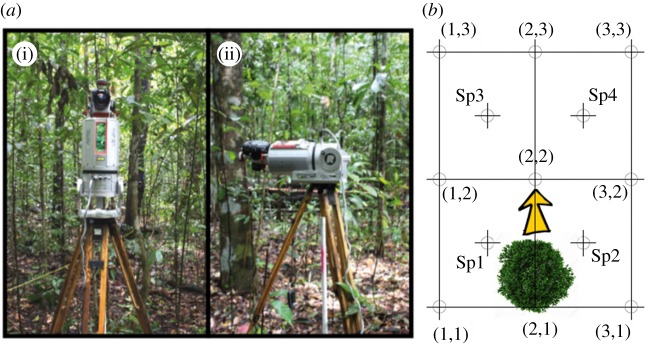
(*a*) Riegl VZ-400: upright position (i) and tilted position (ii). (*b*) Scanning configuration. The 13 scanning positions are set around the predicted falling direction of the tree, on a 3 × 3 grid with four additional interleaved positions.

TLS data were collected using a Riegl VZ-400 (figure 1*a*). The instrument has a rate up to 122 000 measurements per second and a measurement range up to 600 m. It offers a 360° horizontal and 100° vertical field of view and uses a Class 1 near infrared laser beam.

To construct full hemispherical scans, data were acquired using both upright and tilted instrument positions. There were 13 upright scans and 13 tilted scans for each of the two survey sessions (pre-harvest and post-harvest), for a total of 52 scans. The scanning positions were marked with reflectors following the configuration shown in figure 1*b*. This was done to ensure that the same area is scanned before and after tree harvest.

#### 2.2.2. Unmanned aerial vehicle data collection

For this fieldwork, airborne digital photographs were collected using a small multi-rotor UAV (Aerialtronics Altura AT8) equipped with a HYMSY (Hyperspectral Mapping System) sensor. HYMSY is a lightweight (2.0 kg) hyperspectral pushbroom system developed for small UAVs that consists of a custom-made pushbroom spectrometer, a GPS inertial navigation system unit, and a Panasonic GX1 camera [32].

To create the canopy model, images from the Panasonic GX1 camera were used. The camera was triggered once every 2 s, with the focus of the lens fixed to infinity. The mean height of the waypoint flights varied between 50 and 70 m above ground level (appendix A). The hyperspectral data of HYMSY were not used for analysis in this study.

### 2.3. Pre-processing of the collected data

**Figure 2.**
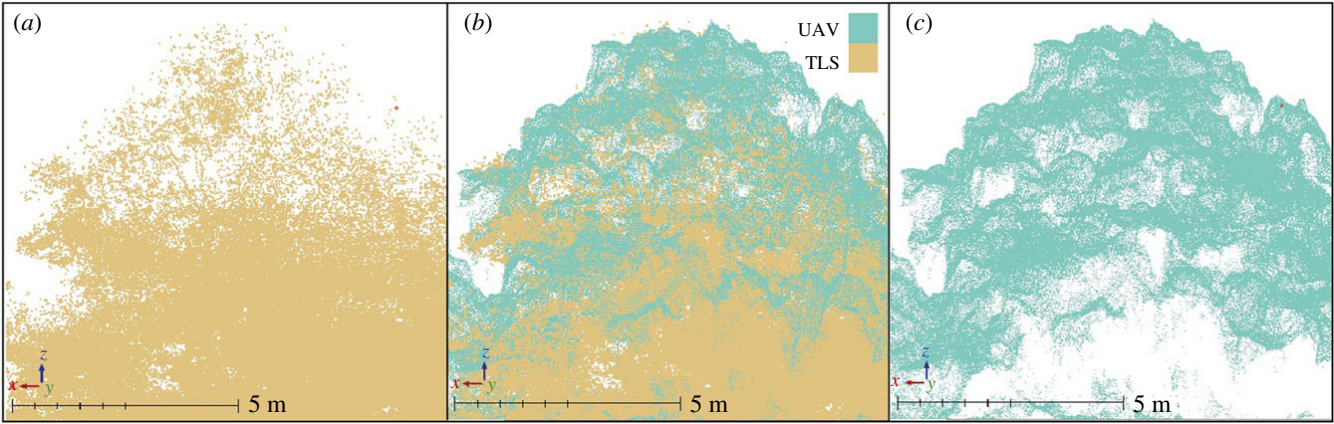
Close-up on the canopy of one emergent tree: TLS point cloud (*a*), TLS and UAV aligned point clouds (*b*), UAV point cloud (*c*).

Using the reflectors and multistation adjustment, the point clouds from each TLS scan position were co-registered, following the protocol described in Gonzalez de Tanago *et al*. [42]. To obtain a point cloud from the aerial photographs, the SfM technique was used in combination with dense stereo-reconstruction techniques. The photographs taken above a certain flight altitude were selected and converted to 8 bit-tiff format. For the rest of the procedure, Agisoft PhotoScan Professional v. 1.1.2 was used (appendix A). First, photographs were aligned, by applying the SfM technique. To do this, the SfM uses algorithms such as SIFT [43] to detect image feature points and matching them between images. A bundle block adjustment was then performed on the matched features to identify the three-dimensional position and orientation of the cameras, their internal calibration and the XYZ location of each feature in the photograph, resulting in a sparse point cloud. Lastly, the majority of geometric scene details are built, by applying a dense, multi-view stereo-reconstruction on the aligned image set [44]. Then, the TLS and UAV point clouds were co-registered (figure 2), and the TCMs were constructed by selecting the highest point within a 30 cm grid cell, converting the surface to raster format for further analysis.

### 2.4. Data analysis

#### 2.4.1. Forest structure features influencing the terrestrial laser scanning and unmanned aerial vehicle top of canopy models

**Figure 3.**
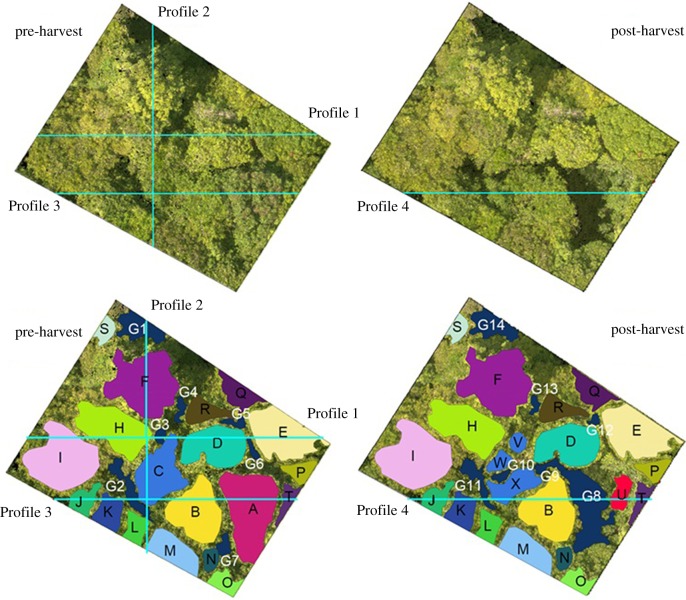
Selected cross-section lines (upper). Profiles 1 and 2 cover most of the plot. Profiles 3 and 4 are over the harvested tree. Delineation of analysed gaps (G1, G2, … , G14) and trees (A, B, … , X).

To determine whether there are differences between the TLS and UAV TCMs a difference raster was calculated, by subtracting the value of each pixel of the TLS TCM raster from the corresponding pixel of the UAV TCM raster. Further, four cross sections were analysed in detail to determine whether there are spatial patterns in the differences between the TLS and the UAV TCMs. The sampling strategy for the cross sections (figure 3) was designed to cover most of the plot area (Profiles 1 and 2), to cross the harvested tree (Profile 3) and to cross the gap left by the harvested tree (Profile 4).

The heights in the profile graphs are relative to the local coordinate system defined by the position of the TLS device in the first scan position, and the values should not be mistaken for tree heights. The horizontal origin of the graphs is the beginning of the transect (left for Profile 1, Profile 3 and Profile 4; top for Profile 2).

It is expected that the largest differences between the TCMs occur in canopy gaps, as Lisein *et al*. [36] suggest that ‘fine-scale gaps’ are not correctly reconstructed. Therefore, all areas representing canopy gaps were evaluated in more detail, and the mean height difference (m) and the percentage of area having a positive height difference (%) were calculated.

#### 2.4.2. Forest change detection

To assess the sensitivity of the two modelling technologies to forest change, a difference raster was computed, by subtracting the post-harvest TCM from the pre-harvest TCM for both the TLS and the UAV data. For the gaps created by the fall of the harvested tree (gaps G8, G9, G10 and G11; figure 3), and for the trees emerged from the understory (trees U, X, W and V; figure 3), the mean height difference (m) and the standard deviation (m) were calculated using the values of all pixels delineated by the contour of the gap or tree (figure 3), for both the TLS and the UAV TCMs.

#### 2.4.3. Sensitivity over repeated surveying sessions

To assess if TLS and airborne SfM derived point clouds render the same TCM over repeated surveying sessions, the difference raster computed above was analysed on the areas not affected by the fall of the trees (figure 3).

For each tree or gap identified in the plot, that was not affected by the harvesting of the crop tree, the mean height difference (m) and the standard deviation (m) were calculated using the extent of the object on the pre–post difference raster, for both the UAV TCMs and the TLS TCMs.

## 3. Results

### 3.1. Forest structure features influencing the terrestrial laser scanning and unmanned aerial vehicle top of canopy models

**Figure 4.**
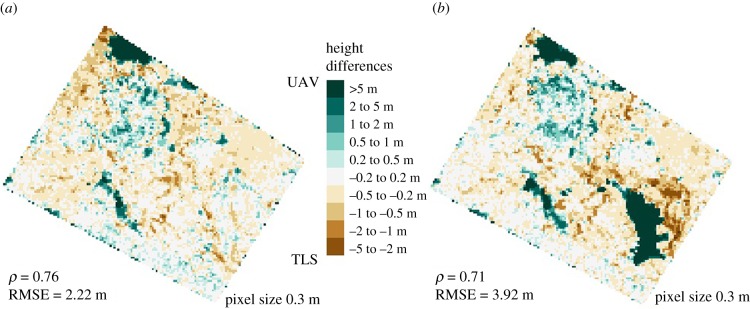
Height differences (UAV minus TLS) between the pre-harvest TCMs (*a*) and post-harvest TCMs (*b*). Positive values (teal) show a higher UAV canopy model, especially in gap areas.

The TCMs from the TLS and the UAV match each other within 0.5 m in 77% (pre-harvest) and 71% (post-harvest) of the pixels (figure 4). On average, on the pre-harvest plot, the UAV TCM is +0.20 m above the TLS TCM. However, the distribution of the difference is uneven, as the 99th percentile of the height difference is +11.42 m (UAV above TLS) while the 1st percentile is just –1.41 m. This indicates that in the places where the UAV TCM is higher than the TLS TCM, the differences in height estimation are large. The same patterns can be observed for the post-harvest TCMs, where, on average, the UAV TCM is +0.76 m above the TLS TCM, with the 99th percentile of the height difference being +21.10 m and the 1st percentile being −2.20 m.

**Figure 5.**
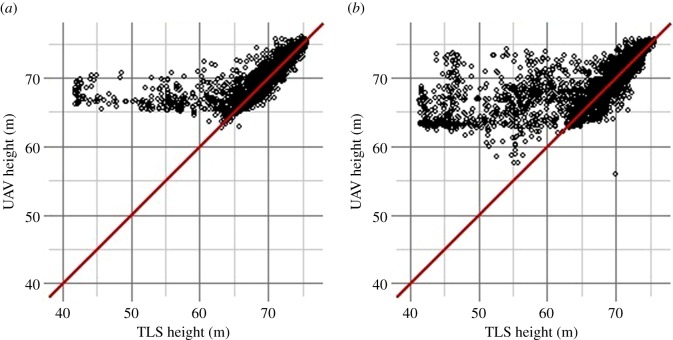
Canopy heights (ground at 41 m) and identity line (red). Most points are concentrated on the identity line, suggesting that both TLS and UAV model the canopy similarly. The outliers are biased towards overestimating UAV heights relative to TLS heights, for both (*a*) pre-harvest and (*b*) post-harvest sessions.

In areas where the canopy is tall (figure 5), the UAV TCM and the TLS TCM match well. However, the UAV TCM did not model low areas well thus it overestimated the height in gap areas. This overestimation is emphasized even more in the post-harvest scatterplot (figure 5) because the harvest of the crop tree created a gap in the canopy, meaning there are more low height areas in the post-harvest plot than in the pre-harvest one.

**Figure 6.**
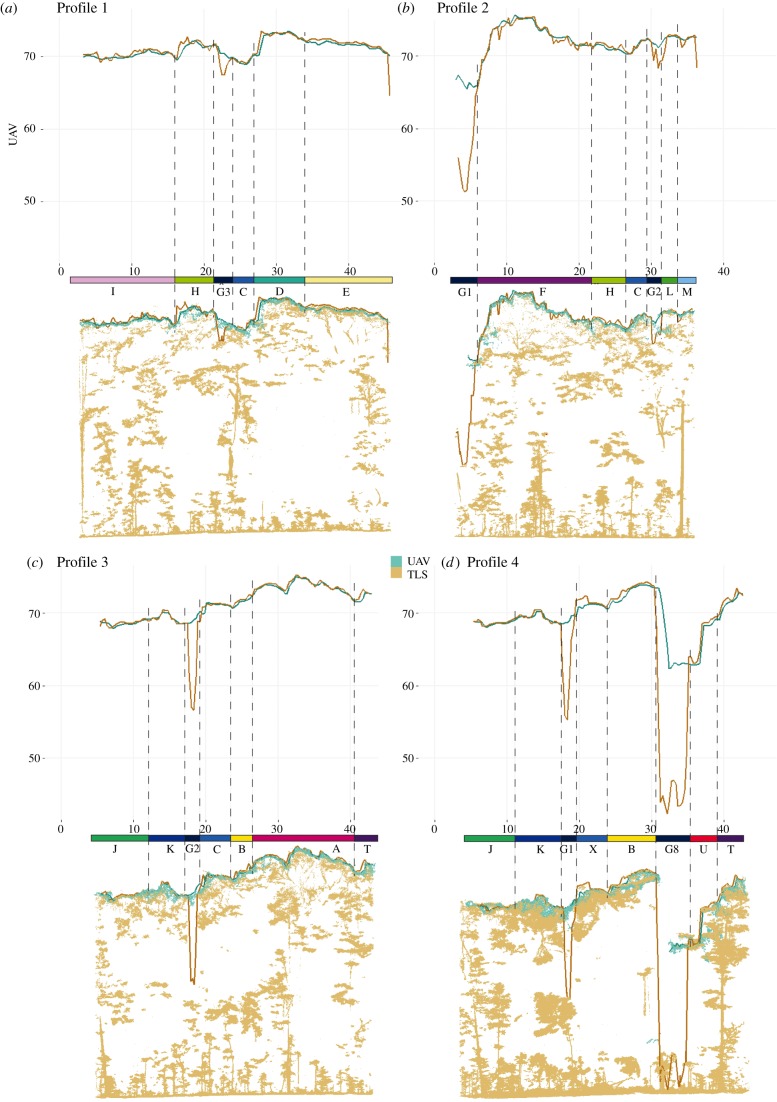
Cross-sections of the UAV and TLS modelled trees (A, B, … , X) and gaps (G1, G2, … , G14), with the TCM profiles plotted on top for each profile (ground at 41 m). The UAV and TLS model trees similarly, while UAV tends to relatively overestimate gap heights.

The UAV and the TLS show differences in TCMs, particularly in areas where canopy gaps exist (figure 6). While the small canopy gaps (G3, figure 6*a*; G2, figure 6*b* and figure 6*c*; G11, figure 6*d*) are completely missed by the UAV TCM, the deeper and larger ones (G1, figure 6*b*; G8, figure 6*d*) are underestimated by the UAV TCM.

To confirm the pattern observed, all gaps identified in the orthophotos illustrated in figure 3 were evaluated. The height difference between the UAV and TLS TCMs was calculated as a raster with the extent of each canopy gap. In all gaps, the UAV TCM is on average higher than the TLS TCM (mean height differences between 0.19 and 13.83 m). Out of 14 gaps, seven have a positive height difference on more than 85% of the area, three of the gaps have a positive height difference on more than 70% and three have more than 50% of the area where the UAV TCM is higher. Gap G6 is the only one that has a positive height difference for less than 50% of the area.

### 3.2. Forest change detection

**Figure 7.**
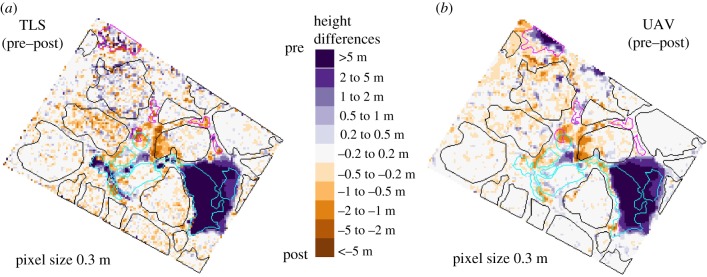
Difference (pre-harvest minus post-harvest) between the TLS TCMs (*a*) and UAV TCMs (*b*), with trees and canopy gaps affected by the harvest (cyan) and unaffected trees (black) and gaps (magenta). Owing to the overestimation of gap heights, not all affected gaps are captured by UAV. The TLS pre–post raster appears noisier because of the TLS TCM high sensitivity to the scanning position.

The harvest of the tree is clearly detected by both the TLS and the UAV TCMs. In both the TLS pre–post difference (figure 7*a*) and UAV pre–post difference (figure 7*b*), a big positive difference (colour purple) can be observed between the pre-harvest survey session and the post-harvest survey session.

**Figure 8.**
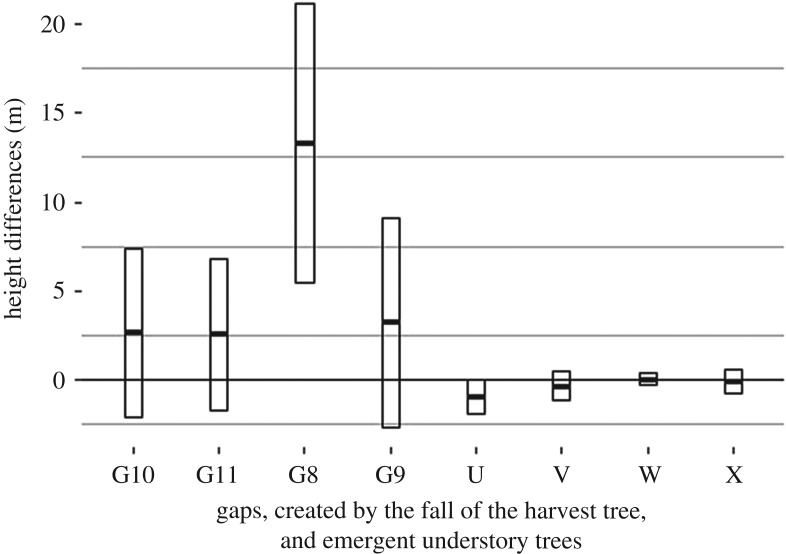
Mean (*m*) and standard deviation (*±m*) of change differences (TLS minus UAV change). Changes are similarly captured for trees, while UAV underestimates gap depths, especially for gap G8, that reaches the ground.

The depth of the canopy gaps, created by the harvesting of the tree, however, differs between the two modelling technologies (figure 8) with the UAV underestimating the depth. The biggest difference can be observed in gap G8 (RMSE of 15.43 m). Nevertheless, figure 8 also shows that the understory trees that remained in the canopy gap created by the harvest of the crop tree are similarly modelled.

### 3.3. Sensitivity over repeated surveying sessions

Although considerably smaller, differences between the TCMs rendered at different points in time exist not only in the area affected by the harvest of the crop tree, but also in the rest of the plot (figure 7).

For all trees, the RMSE of the height differences between the initial survey session (pre-harvest) TLS TCM and the repeated survey session (post-harvest) TLS TCM is between 0.21 and 1.21 m. For the UAV TCM, the RMSE of the height differences between the initial and repeated survey sessions is between 0.11 and 0.63 m. For gaps, the RMSE of the height differences between the initial TLS TCM and the repeated TLS TCM was between 1.02 and 2.48 m, while the RMSE of the height differences between the initial and repeated UAV TCM is between 0.14 and 3.05 m.

**Figure 9.**
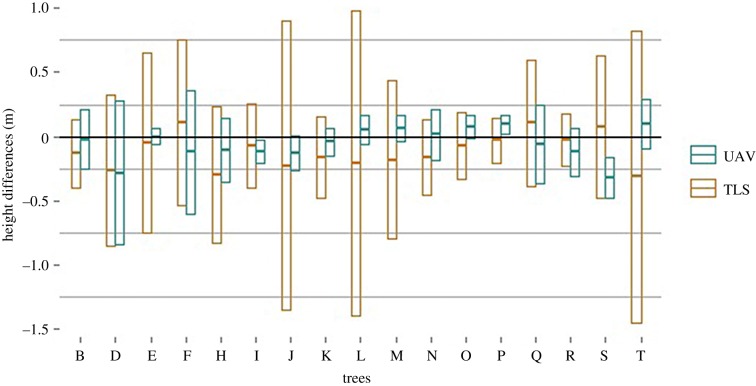
Mean and standard deviation of height differences (pre-harvest minus post-harvest) calculated on UAV TCMs and on TLS TCMs, for pixels of each tree. The standard deviations of TLS height differences are larger because of the high sensitivity of TLS models to the initial scanning positions.

**Figure 10.**
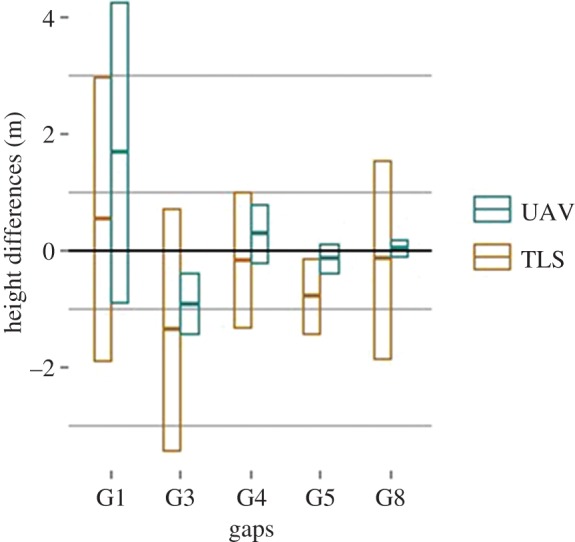
Mean and standard deviation of height differences (pre-harvest minus post-harvest) calculated on UAV TCMs and on TLS TCMs, for pixels of each gap.

These results indicate that the TCMs rendered from UAV data, acquired over the same area, at different moments, are more similar than TCMs rendered from TLS data, acquired over the same area, at different moments. This pattern is illustrated in figures 9 and 10, i.e. the height difference between the initial and repeated TLS TCMs is more spread around the mean than the height difference between the initial and repeated UAV TCMs.

Our analysis shows that TCMs rendered from TLS- and UAV-derived point clouds are sensitive to the survey set-up, and differ slightly over repeated surveying sessions.

## 4. Discussion

### 4.1. Top of canopy models

In our study, the TLS and the UAV TCMs were similar. Based on a literature review, which suggested that in tropical forests the TLS might not be able to reach the top of canopy due to the effect of occlusions [3,4,6,8], a bigger difference between the TLS and UAV TCMs was expected than was seen. Furthermore, it was expected that the taller the trees are, the bigger the difference, because it is more likely that the TLS would not to capture the top of it. However, a high positive Pearson correlation coefficient (0.76 for pre-harvest TCMs and 0.71 for post-harvest TCMs), and a relatively small RMSE (2.20 m for pre-harvest TCMs and 3.92 m for post-harvest TCMs), show that the TLS and the UAV TCMs are very similar. This may be due the very dense configuration of the TLS scanning positions used for this study and the multiple return capabilities of the scanner. For other experimental settings, instruments, or subtypes of tropical forests, the outcomes could be different.

Our results are comparable to those found in the literature. Lisein *et al*. [36] obtained a correlation of 0.86 between the TCMs extracted from their photo canopy height model (CHM) and from the ALS CHM, using a window size of 20 × 20 m for an area of 197.7 ha. Aside from the fact that Lisein *et al*. [36] compared UAV-SfM with ALS and not UAV-SfM with TLS, as in this study, the lower correlation obtained here can be explained by the different window size (20 × 20 m, compared to our 30 × 30 cm), and by the fact that they did not include the areas that had a vegetation height lower than 2 m in the computation of the correlation. Even a small increase in window size in our study increases the correlation between the two TCMs (e.g. for a 1 × 1 m pixel size, the correlation is 0.84). Moreover, the correlation would have definitely improved, especially for the post-harvest, if areas that had a height lower than 2 m would not have been taken into account.

### 4.2. Forest structure features

This study determined that canopy gaps strongly influence how the top of canopy is modelled from UAV and TLS point clouds. In areas where canopy gaps occur, the UAV TCM overestimates canopy height. These findings are consistent with Nayar & Kanade [45] who observe that in concave surfaces points reflect light between themselves and the shape cannot be accurately recreated from stereo restitution. Another study conducted in a mixed forest in Belgium by Lisein *et al*. [36] found that ‘fine-scale gaps’ are not correctly reconstructed by image matching due to the problem of ‘dead-ground’, caused by shadow cast in dense canopy cover. In addition, Dandois & Ellis [35] mention that if the understory is brightly illuminated, point recognition using computer vision is enhanced, producing deeper and denser understory points. The fact that canopy gaps are not accurately modelled from the UAV-derived point clouds makes this technology less suitable for modelling top of canopy for gap-phase regeneration, impacts of logging, light penetration, snow melting and canopy rain interception.

### 4.3. Forest change detection

Both UAV-SfM and TLS have been used to monitor change. TLS has been used to monitor various deformations (land, bridges, other structures) [46–48], while both have been used to monitor landslides [37,49]. While in these studies, accuracies of the order of centimetres are obtained for both instruments, it is important to keep in mind that the surveyed surfaces (mostly bare soil or pastures with no trees) are static at the moment of the survey, with no objects to cause occlusions. This was not the case for the tropical forest surveyed for this research.

The understory trees (U, X, W and V) that emerged in the canopy gap created by the harvest of the crop tree were detected by both the TLS and the UAV TCMs. As expected, the trees were more accurately modelled than the newly created gaps. Although the UAV-SfM does not model gaps well, making its usage in monitoring gap-phase regeneration limited, the change produced by the harvest of the crop tree is detectable (figure 7) and an indication that, despite not being the most precise, UAV TCMs can be used to estimate effects of logging.

### 4.4. Sensitivity over repeated surveying sessions in tropical forest

The main cause for differences between the pre-harvest and the post-harvest TLS TCMs is occlusion. Although the scanning positions of the TLS were marked in the field, it is unlikely that the instrument had exactly the same position and height between the survey sessions. This means that the laser beam hits other branches and passes through other gaps in the canopy than in the previous session, causing a change in the way canopy is modelled. Furthermore, differences may be caused by wind. It did not seem windy at the moment of scanning, but the presence of wind in the upper canopy cannot be discarded.

Differences between the pre-harvest and the post-harvest UAV TCMs occur, because images were taken from flights that had different flight paths. The pre-harvest plot was photographed with the UAV starting from a road located southwest of the plot, while the post-harvest data were acquired with the UAV starting from a road located northeast from the plot. This difference in flight path is accentuated when rendering the understory in a canopy gap. Although the flights in this research followed the same waypoints above the plot, some differences due to the view angle were noticed, as some GCP reflectors placed in canopy gaps were not captured in the pre-flight, while they were captured in the post-harvest flight.

The spotty pattern of the TLS (figure 7*a*), compared to the smooth one of the UAV (figure 7*b*), appears in areas where trees do not have a continuous top layer of leaves. All the small peaks shown in the TLS graphs for trees F, H and I are thin branches that the TLS captures and that are smoothed in the UAV-SfM processing. This effect is also described by Lisein *et al*. [36], who stated that trees with numerous and abrupt fine-scale peaks and gaps in the outer canopy are more affected by the smoothing effect induced by the dense-matching. In their study, Fritz & Koch [50] also noticed that thinner branches remain undetected by the UAV-SfM process. The smoothness of the UAV TCM may be pronounced by the option of ‘aggressive depth filtering’ when creating the dense point cloud.

### 4.5. General considerations

For some purposes, such as calculating surface roughness for atmosphere–land interaction models, or modelling light penetration, the three-dimensional model of the top of canopy alone is useful; nevertheless most studies also use canopy height estimates as inputs. To derive the CHM, a digital terrain model (DTM) must be subtracted from the TCM. TLS has the advantage in that it produces a detailed DTM, whereas the UAV-SfM cannot penetrate the vegetation to reach the ground and so cannot render a DTM directly. Particularly in high slope areas, the only possibility of rendering a DTM is using UAV-SfM.

Both methods have their pros and cons in terms of operability. While the UAV used in this study had a limited flight time of about ­6 min that restricted the study area to the vicinity of the lift-off site, other UAVs (such as the fixed-wing used by Puliti *et al*. [38]) can fly up to 60 min. Likewise, TLS can also have a limited study area, because the instrument itself is heavy (approx. 10 kg), and a lot of additional equipment is needed, which constrains the area of interest to the vicinity of an access road. In the tropical forest of Guyana, in November, the biggest impediment regarding weather conditions is rain. Neither the TLS nor the UAV can acquire data while it is raining, firstly because water may damage the instruments, and secondly because water droplets absorb LiDAR radiation and appear in the images taken from the UAV, affecting the creation of the three-dimensional point cloud. Wind is also undesirable, because movement of the tree branches lowers the accuracy of the canopy model. Moreover, high wind increases the safety risks of operating the UAV. Both instruments can acquire data in full cloud cover conditions. Moreover, in full sun conditions the shadows are strong and clearly visible, causing gaps to be less illuminated and difficult to accurately reconstruct from UAV imagery. In overcast conditions, the shadows are less present, and shadowed areas should give better models as the camera exposure time can suit imaging those better. While a UAV equipped with a consumer digital camera can be relatively inexpensive (typically around 4000€ [35]), and is even available for around 300€ [51], a TLS is much more expensive, around 100 000€ [52].

## 5. Conclusion

Most differences between UAV and TLS collected data were known before this study. The present research fills a knowledge gap about the viability of using these technologies for modelling the top of canopy in tropical forests. Our results show that the UAV and TLS TCMs are largely similar, especially in an undisturbed canopy. The main differences between UAV and TLS TCMs are that the former is smoother and less precise over gaps, while the latter is more detailed, but also more sensitive to changes in the experimental set-up.

## Data accessibility

All data needed to evaluate the conclusions of the study are present in the paper. Additional access to the raw data, processed data and R scripts can be provided by the authors upon request.

## Appendix A

See tables 1–3.

**Table 1.** UAV imagery resolution.

flight altitude (m)	canopy height (m)	Panasonic GX1		
		width (m) (cross-path)	height (m) (along path)	ground sample distance (cm) (top of canopy for this study)
50	30	24	18	0.52
70	30	48	36	1.0

**Table 2.** UAV processing parameters in Agisoft PhotoScan Professional v. 1.1.2.

action	parameter value	selected value
align photos	accuracy	high
	pair preselection	disabled
build dense cloud	quality	high
	depth filtering	aggressive

**Table 3.** Accuracy of photogrammetric processing.

	RMSE (cm) of GCPs	
	before camera optimization	after camera optimization
pre-harvest flight	19.6	4.1
post-harvest flight	24.2	7.6
